# Inhibition of KCa3.1 Channels Suppresses Atrial Fibrillation via the Attenuation of Macrophage Pro-inflammatory Polarization in a Canine Model With Prolonged Rapid Atrial Pacing

**DOI:** 10.3389/fcvm.2021.656631

**Published:** 2021-05-31

**Authors:** Shanqing He, Youcheng Wang, Yajun Yao, Zhen Cao, Junkui Yin, Liuliu Zi, Huiyu Chen, Yuntao Fu, Xi Wang, Qingyan Zhao

**Affiliations:** ^1^Department of Cardiology, Renmin Hospital of Wuhan University, Wuhan, China; ^2^Cardiovascular Research Institute of Wuhan University, Wuhan, China; ^3^Hubei Key Laboratory of Cardiology, Wuhan, China

**Keywords:** atrial fibrillation, KCa3.1 channel, macrophage, inflammation, canine

## Abstract

**Aims:** To investigate the role of KCa3. 1 inhibition in macrophage pro-inflammatory polarization and vulnerability to atrial fibrillation (AF) in a canine model with prolonged rapid atrial pacing.

**Materials and Methods:** Twenty beagle dogs (weighing 8–10 kg) were randomly assigned to a sham group (*n* = 6), pacing group (*n* = 7) and pacing+TRAM-34 group (*n* = 7). An experimental model of AF was established by rapid pacing. TRAM-34 was administered to the Pacing+TRAM-34 group by slow intravenous injection (10 mg/kg), 3 times each day. After 7 days of pacing, the electrophysiology was measured *in vivo*. The levels of interleukin-1β (IL-1β), monocyte chemotactic protein-1 (MCP-1), tumor necrosis factor-α (TNF-α), CD68, c-Fos, p38, and NF-κB p65 in both atriums were measured by Western blotting, and the levels of inducible nitric oxide synthase (iNOS) and arginase1 (Arg-1) were measured by real-time PCR. Macrophage and KCa3.1 in macrophage in the atrium were quantized following double labeled immunofluorescent.

**Results:** Greater inducibility of AF, an extended duration of AF and lower atrial effective refractory period (AERP) were observed in the pacing group compared with those in the sham group. Both CD68-labeled macrophage and the expression of KCa3.1 in macrophage were elevated in the pacing group and inhibited by TRAM-34, led to higher iNOS expression, lower Arg-1 expression, elevated levels of IL-1β, MCP-1, and TNF-α in the atria, which could be reversed by TRAM-34 treatment (all *P* < 0.01). KCa3.1 channels were possibly activated via the p38/AP-1/NF-κB signaling pathway.

**Conclusions:** Inhibition of KCa3.1 suppresses vulnerability to AF by attenuating macrophage pro-inflammatory polarization and inflammatory cytokine secretion in a canine model with prolonged rapid atrial pacing.

## Introduction

Atrial fibrillation (AF) can give rise to serious health consequences in humans, although the mechanism by which it is initiated or maintained is not entirely clear. It has been demonstrated in a number of studies that electrical remodeling is an important modifier that affects an individual's susceptibility to AF, and there is now a consensus that changes to ion channels and intercellular interstitial connections represent the principal manifestations of electrical remodeling (1, 2). However, recent research in electrophysiology suggests that macrophages and inflammation also play an important role in electrical remodeling. For example, TNF-α was found to participate in the pathogenesis of AF, probably via a decrease in T-type current density in atrial myocytes via the down-regulation of channel protein expression and the loss of channel functionality (3). It was also demonstrated that macrophages may inhibit the expression of atrial myocyte quaking protein via the secretion of IL-1β (4). Such evidence possibly indicates that macrophages and inflammation play a role in the pathophysiology of AF.

KCa3.1 is a calcium-activated K^+^ channel that opens only in response to increased cytosolic calcium which has been reported to have a regional and functional distribution within the heart (5, 6). Previous studies suggest that KCa3.1 may be of particular interest in arrhythmia. TRAM-34, a KCa3.1 inhibitor, has been shown to prolong the PR interval and slow the heart rate (7, 8). In previous studies we demonstrated that KCa3.1 inhibited by TRAM-34 completely inhibited the induction of AF and prolonged the decline in AERP in canines with rapid atrial pacing (9). In addition, upregulation of the KCa3.1 channel was shown to be associated with vulnerability to atrial fibrillation in a canine model of acute stroke (10). Although there are prospects for the treatment of AF via control of KCa3.1, the mechanism of action of KCa3.1 in AF remains unclear. It has been reported that the inhibition of KCa3.1 reduces macrophage activation and levels of cytokines in the brain, such as IL-1β, suggesting a potential novel association between KCa3.1 and AF (11). In the present study, the role of KCa3.1 inhibition in AF was investigated via attenuation of macrophage pro-inflammatory polarization and inflammation in a canine model of AF with prolonged rapid atrial pacing.

## Materials and Methods

The study was approved by the animal studies subcommittee of our institutional review board and was in compliance with the guidelines of the National Institutes of Health for the care and use of laboratory animals.

### Animal Model Preparation

Beagles (males, weighing 8–10 kg, 15 ± 2 months old) were maintained and bred in the Animal Experimental Center of Renmin Hospital at Wuhan University. Twenty beagles were randomly assigned to three groups. The control group consisted of six canines in which pacemakers were implanted under sterile conditions without atrial pacing. The pacing group consisted of seven canines in which implanted pacemakers provided continuous atrial pacing (450 beats/min) for 7 days. The pacing+TRAM-34 (KCa3.1 blocker, MedChemExpress, USA) group consisted of seven canines in which the same pacing model was induced but with the administration of TRAM-34 by slow intravenous injection (10 mg/kg) three times each day. The vehicle that was used for the slow iv infusion was mixed by adding 10% DMSO, 40% PEG300, 5% Tween-80, 45% saline in sequence and the pacing group also was infused with vehicle.

Each canine received an intramuscular injection of 25 mg/kg ketamine sulfate prior to premedication with pentobarbital sodium (ASPEN Biotechnology Co, China), after which they were intubated and ventilated with ambient air supplemented with oxygen from a respirator (MAO01746, Harvard Apparatus Holliston, USA). Continuous ECG monitoring was performed.

### Cardiac Pacemaker Implantation

An atrial electrode was placed in the right atrium via the right external jugular vein while viewing with X-ray fluoroscopy. A pouch was created under the subclavian skin in which a high-frequency pacemaker was placed and connected to the electrode via a subcutaneous tunnel. After successful pacing of the right atrium, the electrode was fixed in place and the pouch closed with sutures. Four million units of penicillin were injected intramuscularly for 3 consecutive days after surgery.

### Electrophysiological Measurements

The heart was exposed by forming a pericardial cradle after median sternotomy under anesthesia in each group of dogs. The atrial effective refractory period (AERP) was measured as described previously (12). Multielectrode catheters (Biosense-Webster, Diamond Bar, CA) were secured to allow recording from both the left and right atria (LA/RA). All recordings were displayed on a computerized electrophysiology system (Lead 7000, Jinjiang Inc., China). An S1S1 programmed stimulus method (120-ms, 100-ms, and 75-ms cycle length, 5 s each, performed in triplicate for each frequency) was used to assess the inducibility and duration of AF. AF was defined as an irregular atrial rate >500 bpm lasting for more than 5 s.

### Double Labeled Immunofluorescence

Following electrophysiological measurement, the atrial tissues were quickly removed and fixed in 4% paraformaldehyde, embedded in paraffin then sectioned to 5 microns. To identify KCa3.1 and the pro-inflammatory M1 macrophages, the primary KCa3.1 antibody (bs-6675R, Bioss, 1:200) and CD68 antibody (KP1) (sc-20060, Santa Cruz, 1:50) was added to the sections overnight at 4°C. Then, the sections were incubated with the secondary antibody Fluorescein (FITC)–conjugated Affinipure Goat Anti-rabbit IgG (Aspen, AS1110; 1:50) and Fluorescein (CY3)–conjugated Affinipure Goat Anti-mouse IgG (Aspen, AS1111; 1:50) and cell nuclei by DAPI. The results were analyzed using Image-Pro Plus 6.0 software. In each group, four atrial samples from different individuals were randomly selected and used to calculate the mean number of macrophage and quantificat KCa3.1 expression in macrophage in three different fields at 400x magnification. The mean optical density of KCa3.1 in macrophage = Sum of KCa3.1 optical density values in CD68 marked area/CD68 marked area.

### Real-Time PCR

The expression of inducible nitric oxide synthase (iNOS) and arginase1 (Arg-1) in the atria was measured by real-time PCR. Total RNA was isolated from atrial samples using Tripure extraction reagent (ELK Biotechnology, China) in accordance with the manufacturer's protocol. cDNA was synthesized using an EntiLink™ first-strand cDNA synthesis kit (ELK Biotechnology, China). Real-time fluorescent quantitative PCR was performed using a StepOne real-time PCR instrument (Life Technologies, Gaithersburg, MD). Each sample was measured in triplicate using an EnTurbo™ SYBR Green PCR SuperMix kit (ELK Biotechnology, China). Primers used for RT-PCR were: Canine iNOS: 5′-ACCAATACAGGCTCGTGCAG-3′ (forward), 5′-GGGCTGTCTACTACTCGCTCC-3′ (reverse); Canine GAPDH: 5′-GAAGGTCGGAGTGAACGGATT-3′ (forward), 5′-CATTTGATGTTGGCGGGATC-3′ (reverse); Canine Arg-1: 5′-GGCAGAAGTCAAGAAGAACGG-3′ (forward), 5′-CTTTGGCAGATAGGCAAGGAG-3′ (reverse); Canine GAPDH: 5′-GAAGGTCGGAGTGAACGGATT-3′ (forward), 5′-CATTTGATGTTGGCGGGATC-3′ (reverse).

### Western Blotting

Protein identity on membranes was evaluated with primary antibodies against KCa3.1 (Abcam, UK), IL-1β (Abcam, UK), MCP-1 (Abcam, UK), TNF-α (Abcam, UK), CD68 (Abcam, UK), c-Fos (Abcam, UK), p38 (Abcam, UK), or NF-κB p65 (Abcam, UK). Non-specific binding was blocked by adding 5% non-fat dry milk in Tris-buffered saline with Tween 20 (TBST) to membranes for 1 h which were then incubated with primary antibody overnight at 4°C. They were then washed in TBST three times, incubated with a secondary antibody for 1 h at 37°C, then imaged after the addition of Immun-Star horseradish peroxidase substrate. The relative expression levels of proteins was determined using AlphaEasefc 4.0 image analyzer software (AlphaEase FC, USA), by evaluation of the gray density value of each band and comparison with that of GAPDH.

### Statistical Analysis

Values are presented as means ± standard deviation. A two-sample independent student's *t*-test was used to compare the means of two groups. ANOVA combined with a Newman–Keuls test was used to compare the mean values of continuous variables between multiple groups. The significance of differences was further evaluated using a Tukey-Kramer test. All the statistical tests were two-sided, with *p*-values < 0.05 considered statistically significant. All statistical analyses were conducted using SPSS 20.0 software.

## Results

### Electrophysiological Testing and Induction of AF

**Figure 1:**
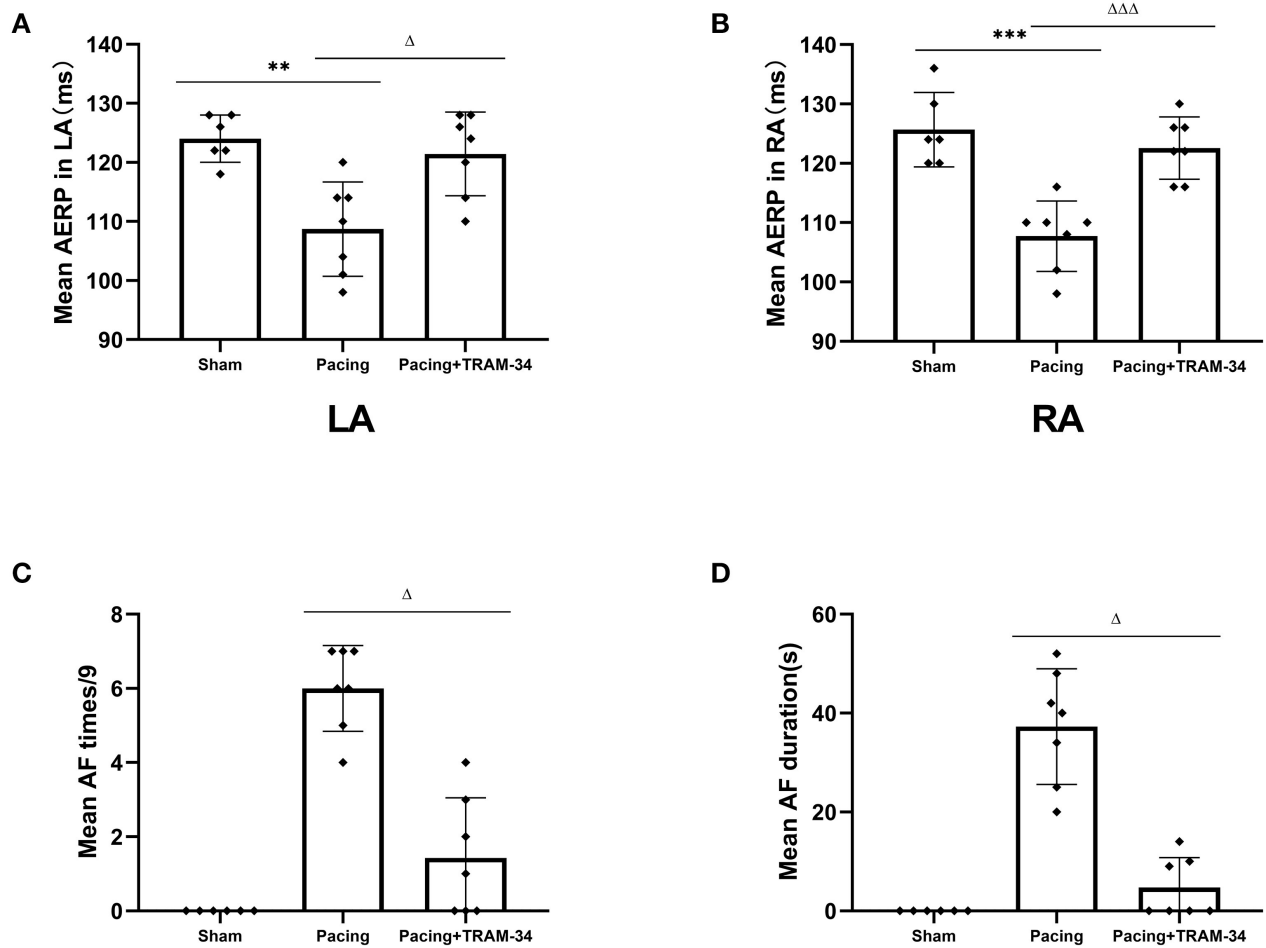
Mean AF inducibility, AF duration and AERP in all groups. **(A,B)** The mean AERP in both LA and RA was substantially greater in the pacing group than in the other two groups, and the pacing+TRAM-34 group was substantially greater than in the sham group. **(C)** The mean AF incidence rate was substantially greater in the pacing group than in the other two groups, and for the pacing+TRAM-34 group it was substantially greater than in the sham groups. **(D)** The mean AF duration was substantially greater in the pacing group than that in the other two groups, and in the pacing+TRAM-34 group it was substantially greater than in the sham group. P** < 0.01 vs. the sham group, P*** < 0.001 vs. the sham group. *P*^ΔΔ^ < 0.01 vs. the pacing group, *P*^ΔΔΔ^ < 0.001 vs. the pacing group. AF, atrial fibrillation; AERP, atrial effective refractory period; RA, right atrium; LA, left atrium.

The AERP at the recording sites was significantly shortened in the pacing group compared with those in the sham group (108.7 ± 8.0 vs. 124 ± 4 ms in the LA, *P* < 0.01, 107.7 ± 5.9 vs. 125.7 ± 6.3 ms in the RA, *P* < 0.001), as shown in Figure 1, and increased when treated with TRAM-34 (108.7 ± 8.0 vs. 121.3 ± 7.1 ms in the LA, *P* < 0.05, 107.7 ± 5.9 vs. 122.7 ± 5.3 ms in the RA, *P* < 0.001). There was no significant difference in AERP in the sham group compared with the pacing + TRAM-34 group in the LA. After 7 days of rapid atrial pacing, the mean AF incidence rate was higher in the pacing group than it was in the sham group (6 ± 1.15 s vs. 0 s, *P* < 0.001), which was reduced by treatment with TRAM-34 (6 ± 1.15 s vs. 1.43 ± 1.62 s, *P* < 0.05). The mean duration of AF was higher in the pacing group compared with that of the sham group (37.29 ± 11.70 vs. 0 s, *P* < 0.001), and reduced when treated with TRAM-34 (37.29 ± 11.70 vs. 4.71 ± 6.08 s, *P* < 0.05). The mean AF incidence rate and the mean duration of AF was higher in the pacing + TRAM-34 group compared with that of the sham group (*P* < 0.001).

### Expression Levels of KCa3.1 Protein in Macrophage and Pro-inflammatory Polarization of Macrophages in the Atria

**Figure 2:**
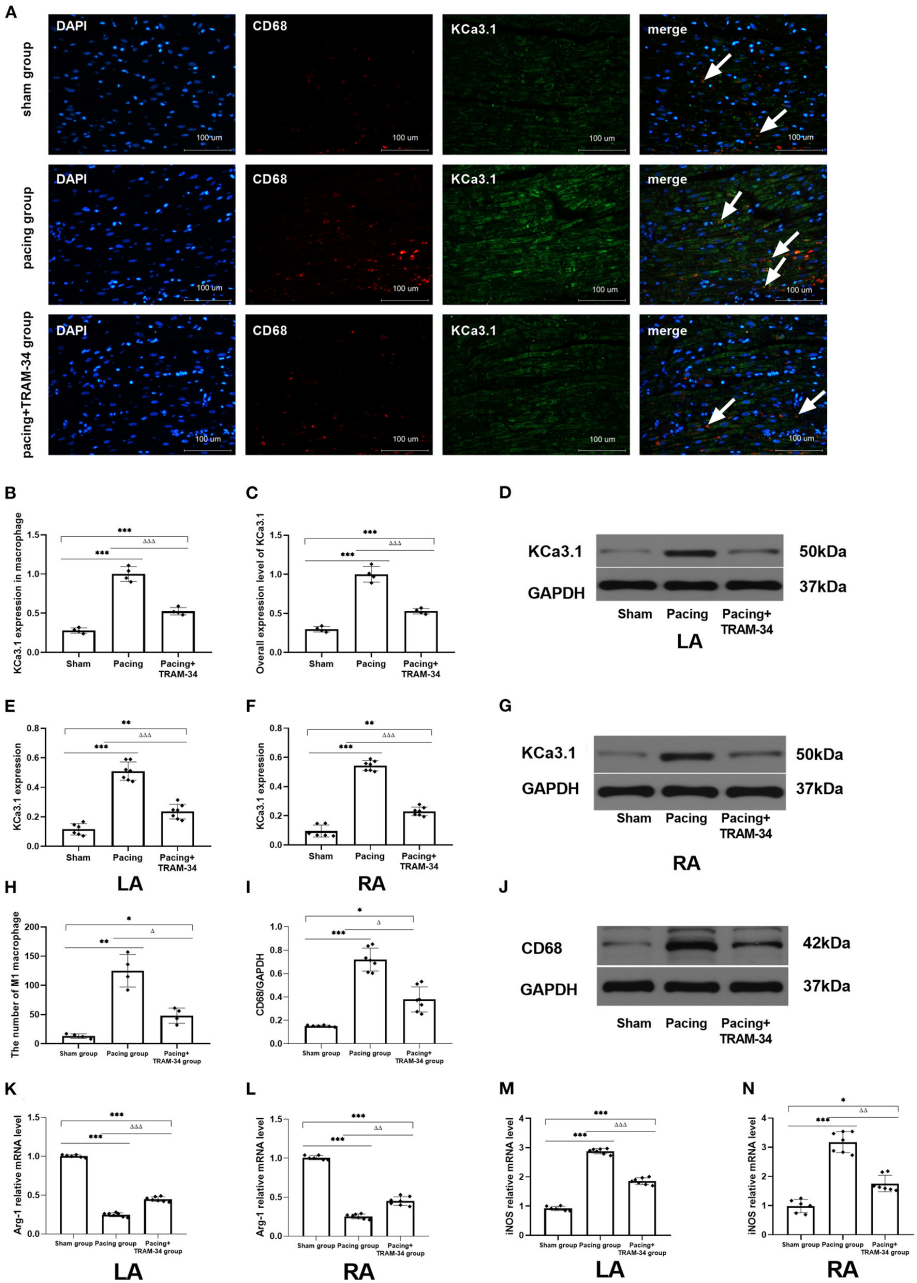
**(A)** Representative atrial sections double labeled immunofluorescence for KCa3.1 and CD68-marked macrophage (while arrow). **(B)** Immunofluorescence analysis indicated that the expression of KCa3.1 in macrophage in the atrium was significantly higher in the pacing group than in the sham group and pacing+TRAM-34 group. **(C)** Immunofluorescence analysis indicated that the overall expression level of KCa3.1 (mainly in cardiomyocytes according to the immunofluorescence figure) in the atrium was significantly higher in the pacing group than in the sham group and pacing+TRAM-34 group. **(D–G)** Western blotting demonstrated that the expression of KCa3.1 in the LA and RA was higher in the pacing group compared with the control and pacing+TRAM34 groups. There was no significant difference in the expression of KCa3.1 between the LA and the RA in the three groups, respectively. **(H)** Quantitative analysis of M1 macrophages in sections of atrial samples (*n* = 4). The number of M1 macrophages was significantly higher in the pacing group than in the sham or pacing+TRAM-34 groups. The number of M1 macrophage in atrium was higher in the pacing+TRAM-34 group than that in the sham group. **(I,J)** The expression of CD68 detected by WB analysis. The relative level of CD68 in the pacing group was markedly higher than in the sham and pacing+TRAM-34 groups. The pacing+TRAM-34 group had a higher level of CD68 in the atrium compared with sham group. **(K,L)** The expression of Arg-1 detected by RT-PCR analysis. The relative level of Arg-1 mRNA in the pacing group was markedly depressed both in LA and RA than in the sham-operated or pacing+TRAM-34 groups. The pacing+TRAM-34 group had a lower level of Arg-1 mRNA in the atrium compared with the sham-operated group. **(M,N)** The expression of iNOS detected by RT-PCR analysis. The relative level of iNOS mRNA in the pacing group was markedly higher in both the LA and RA than in the sham-operated and pacing+TRAM-34 groups. The pacing+TRAM-34 group had a higher level of iNOS in the atrium compared with the sham group (*P* < 0.001). P* < 0.05 vs. the sham group, P** < 0.01 vs. the sham group, P*** < 0.001 vs. the sham group. *P*^Δ^ < 0.05 vs. the pacing group, *P*^ΔΔ^ < 0.01 vs. the pacing group, *P*^ΔΔΔ^ < 0.001 vs. the pacing group. In our experiment, the same GAPDH was used for TNF-α, MCP-1, and the same GAPDH was used for IL-β, p38, c-Fos. Arg-1, Arginase 1; iNOS, Inducible Nitric Oxide Synthase; RA, right atrium; LA, left atrium.

Representative atrial sections double labeled immunofluorescence for KCa3.1 and macrophage are shown in Figure 2. The result provided a demonstration of the following: (1) both CD68-labeled macrophage and the expression of KCa3.1 in macrophage were elevated in the pacing group and inhibited by TRAM-34 (*P* < 0.05). (2) in addition to macrophages, KCa3.1 is also widely expressed in cardiomyocytes. And the overall expression level of KCA3.1 in atria (mainly in cardiomyocytes according to the immunofluorescence figure) increased after atrial pacing. The expression of KCa3.1 protein in the LA and RA was compared in the sham, pacing, and pacing + TRAM-34 groups by Western blotting, as displayed in Figure 2, its expression higher in the pacing group than in the control group (LA: 0.510 ± 0.062 vs. 0.115 ± 0.039, *P* < 0.001; RA: 0.543 ± 0.036 vs. 0.096 ± 0.041; *P* < 0.001), which was reduced by TRAM-34 treatment (LA: 0.543 ± 0.036 vs. 0.096 ± 0.041; *P* < 0.01; RA: 0.543 ± 0.036 vs. 0.214 ± 0.030; *P* < 0.01). There was no significant difference in the expression of KCa3.1 between the LA and the RA for any of the three groups, respectively. Western blotting also demonstrated that the concentration of CD68 was significantly higher in the pacing group than in the sham group which was reduced by treatment with TRAM-34. In addition, the concentration of atrial CD68 was higher in the pacing + TRAM-34 group than in the sham group. Quantification of Arg-1 and iNOS expression was determined by real time PCR for clarification purposes. As shown in Table 1, the relative levels of Arg-1 mRNA in the atria were substantially lower in the pacing group than in the sham group, which was reduced by treatment with TRAM-34. The pacing + TRAM-34 group had a lower level of Arg-1 mRNA in the atria compared with the sham group. The relative levels of iNOS mRNA in the atria were markedly elevated in the pacing group compared with that in the sham group, which was reduced by treatment with TRAM-34. The pacing + TRAM-34 group displayed a higher level of iNOS expression than the sham group.

**Table 1:** Levels of Arg-1 and iNOS in atrial tissue.

	**LA**		**RA**	
	**LA**		**RA**	
	**Arg-1**	**iNOS**	**Arg-1**	**iNOS**
sham group (*n* = 6)	1.003 ± 0.015^ΔΔΔ^	0.923 ± 0.075^ΔΔΔ^	1.007 ± 0.031^ΔΔΔ^	0.987 ± 0.230^ΔΔΔ^
pacing group (*n* = 7)	0.247 ± 0.025	2.880 ± 0.089	0.253 ± 0.031	3.180 ± 0.356
pacing+TRAM-34 group (*n* = 7)	0.447 ± 0.031^***Δ*ΔΔ*^	1.853 ± 0.117^***Δ*ΔΔ*^	0.450 ± 0.057^***ΔΔ^	1.757 ± 0.282^*ΔΔ^

P^*^ < 0.05 vs. the sham group, P^***^ < 0.001 vs. the sham group.

*P^ΔΔ^ < 0.01 vs. the pacing group, P^ΔΔΔ^ < 0.001 vs. the pacing group*.

*Arg-1, Arginase 1; iNOS, Inducible Nitric Oxide Synthase; RA, right atrium; LA, left atrium*.

### Levels of Inflammatory Cytokines in the Atria

**Figure 3:**
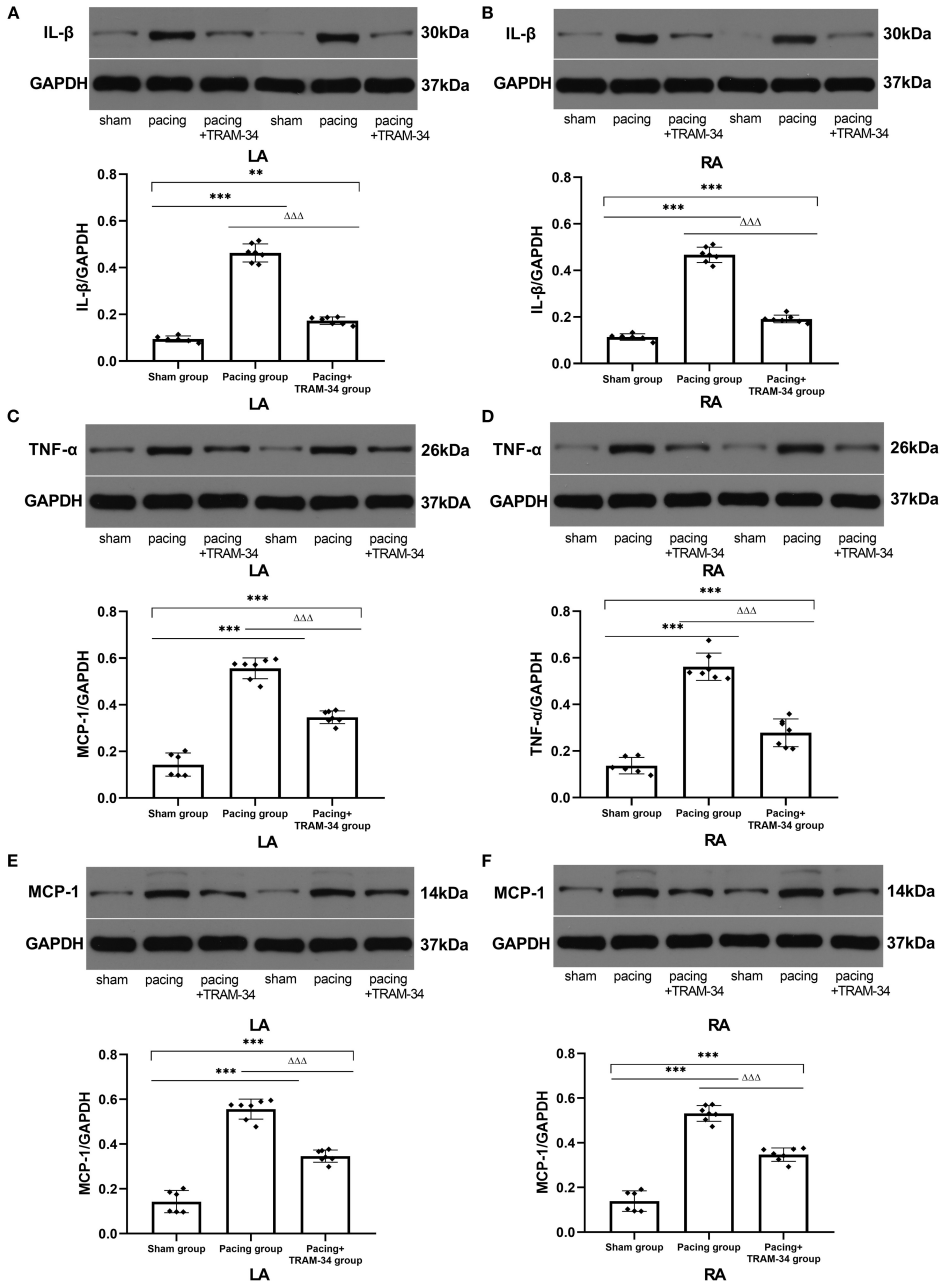
Expression of IL-1β, TNF-α, and MCP-1 in all groups. **(A–F)** Levels of IL-1β, TNF-α, and MCP-1 in both the LA and RA were markedly higher in the pacing group compared with the sham or pacing+TRAM-34 groups. The levels of IL-1β, TNF-α, and MCP-1 in both the LA and RA were higher in the pacing+TRAM-34 group than in the sham group. P** < 0.01 vs. the sham group, P*** < 0.001 vs. the sham group. *P*^ΔΔΔ^ < 0.001 vs. the pacing group. In our experiment, the same GAPDH was used for TNF-α, MCP-1, and the same GAPDH was used for IL-β, p38, c-Fos. IL-1β, interleukin-1 beta; TNF-α, tumor necrosis factor-alpha; MCP-1, monocyte chemotactic protein 1; LA, left atrium; RA, right atrium.

All immunoblot band intensity measurements were normalized to the intensity of the GAPDH band in each sample. As displayed in Figure 3 and Table 2, the levels of IL-1β, MCP-1, and TNF-α in the atria were considerably higher in the pacing group than those in the sham-operated group, which were reduced by treatment with TRAM-34. The levels of IL-1β, MCP-1, and TNF-α in the atria were higher in the pacing+TRAM-34 group than in the sham group.

**Table 2:** Levels of inflammatory cytokines in atrial tissue.

	**LA**			**RA**		
	**TNF-α**	**MCP-1**	**IL-1β**	**TNF-α**	**MCP-1**	**IL-1β**
sham group (*n* = 6)	0.137 ± 0.037^ΔΔΔ^	0.143 ± 0.051^ΔΔΔ^	0.114 ± 0.015^ΔΔΔ^	0.137 ± 0.035^ΔΔΔ^	0.139 ± 0.046^ΔΔΔ^	0.062 ± 0.012^ΔΔΔ^
pacing group (*n* = 7)	0.566 ± 0.062	0.542 ± 0.046	0.468 ± 0.031	0.562 ± 0.059	0.532 ± 0.035	0.468 ± 0.047
pacing+TRAM-34 group (*n* = 7)	0.280 ± 0.058^**Δ*ΔΔ*^	0.346 ± 0.028^***Δ*ΔΔ*^	0.189 ± 0.018^***Δ*ΔΔ*^	0.278 ± 0.060^***Δ*ΔΔ*^	0.347 ± 0.030^***Δ*ΔΔ*^	0.133 ± 0.029^***Δ*ΔΔ*^

P** < 0.01 vs. the sham group, P^***^ < 0.001 vs. the sham group.

*P^ΔΔΔ^ < 0.001 vs. the pacing group*.

*MCP-1, monocyte chemotactic protein 1; TNF-α, tumor necrosis factor-alpha; IL-1β, interleukin-1 beta; LA, left atrium; RA, right atrium*.

### Levels of Signaling Pathway Protein

**Figure 4:**
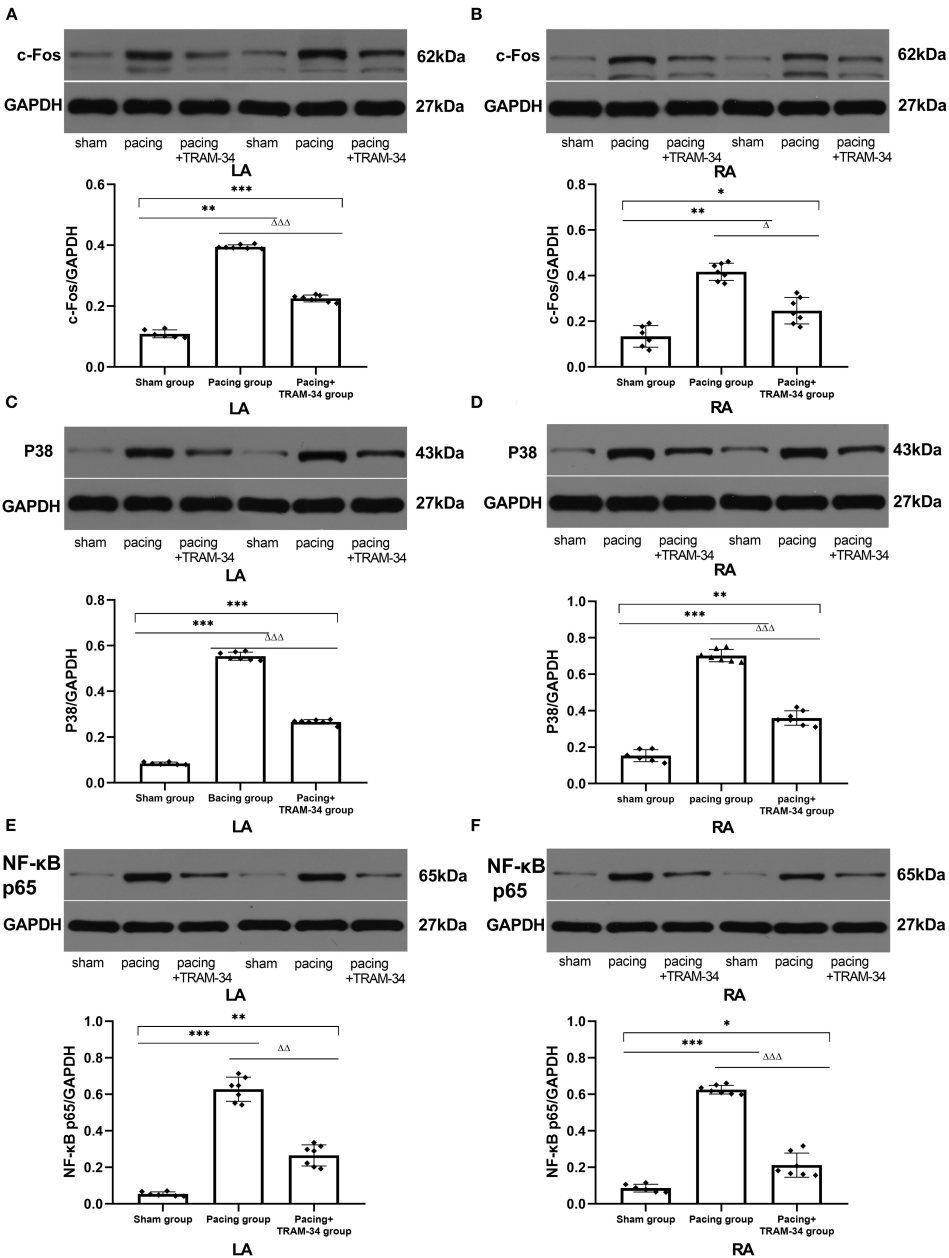
Expression of c-Fos, p38, and NF-κB p65 in all groups. **(A–F)** The levels of c-Fos, p38, and NF-κB p65 in both the LA and RA were markedly higher in the pacing group, compared with the sham or pacing+TRAM-34 groups. The levels of c-Fos, p38, and NF-κB p65 in both the LA and RA were higher in the pacing+TRAM-34 group than in the sham group. P* < 0.05 vs. the sham group, P** < 0.01 vs. the sham group, P*** < 0.001 vs. the sham group. *P*^Δ^ < 0.05 vs. the pacing group, *P*^ΔΔ^ < 0.01 vs. the pacing group, *P*^ΔΔΔ^ < 0.001 vs. the pacing group. In our experiment, the same GAPDH was used for TNF-α, MCP-1, and the same GAPDH was used for IL-β, p38, c-Fos. NF-κB, nuclear factor kappa-B; LA, left atrium; RA, right atrium.

As shown in Figure 4 and Table 3, the expression of c-Fos, p38, and NF-κB p65 in the atria was markedly higher in the pacing group compared with that in the sham group which was reduced by treatment with TRAM-34. The expression of c-Fos, p38, and NF-κB p65 in the atria was higher in the pacing + TRAM-34 group than in the sham group.

**Table 3:** Levels of signaling pathway protein in atrial tissue.

	**LA**			**RA**		
	**P38**	**c-Fos**	**P65**	**P38**	**c-Fos**	**P65**
sham group (*n* = 6)	0.084 ± 0.006^ΔΔΔ^	0.109 ± 0.013^ΔΔ^	0.054 ± 0.011^ΔΔΔ^	0.153 ± 0.033^ΔΔΔ^	0.138 ± 0.048^ΔΔ^	0.086 ± 0.021^ΔΔΔ^
pacing group (*n* = 7)	0.554 ± 0.018	0.394 ± 0.007	0.578 ± 0.157	0.702 ± 0.036	0.417 ± 0.038	0.625 ± 0.024
pacing+TRAM-34 group (*n* = 7)	0.266 ± 0.010^***Δ*ΔΔ*^	0.225 ± 0.011^***Δ*ΔΔ*^	0.265 ± 0.058^**ΔΔ^	0.360 ± 0.040^***Δ*ΔΔ*^	0.246 ± 0.058^*Δ^	0.218 ± 0.066^*Δ*ΔΔ*^

P^*^ < 0.05 vs. the sham group, P^**^ < 0.01 vs. the sham group, P^***^ < 0.001 vs. the sham group.

*P^Δ^ < 0.05 vs. the pacing group, P^ΔΔ^ < 0.01 vs. the pacing group, P^ΔΔΔ^ < 0.001 vs. the pacing group*.

*NF-κB, nuclear factor kappa-B; LA, left atrium; RA, right atrium*.

## Discussion

In the present study, we investigated the role of inhibition of KCa3.1 in macrophage pro-inflammatory polarization in a canine model of AF with prolonged rapid atrial pacing. We provided a demonstration of the following: (1) the expression of KCa3.1 in macrophage increased significantly in the LA and RA of dogs after prolonged rapid atrial pacing, and down-regulated by TRAM-34; (2) inhibition of KCa3.1 channels decreased the vulnerability to AF after prolonged rapid atrial pacing and attenuated macrophage pro-inflammatory polarization and inflammation; (3) the effects of inhibition of KCa3.1 on AF vulnerability are probably associated with macrophage activation and the pro-inflammatory p38 MAPK/AP-1/NF-κB signaling pathway.

Calcium-activated potassium channels can be categorized as high (or large) -conductance calcium-activated potassium channels, intermediate-conductance Ca^2+^-activated K^+^ channels (KCa3.1), also termed SK4, or small-conductance Ca^2+^-activated K+ channels, depending on their conductance level (13). Cardiac automaticity and arrhythmias were recently found to be associated with KCa3.1. In embryonic stem cell-derived cardiomyocytes (ESCCMs), TRAM-34, an alternative blocker of KCa3.1 channels, not only significantly reduced or even stopped the automaticity of human embryonic stem-cell-derived cardiomyocytes, they also decreased the sinus node firing rate (14, 15). In polymorphic ventricular tachycardia patients, TRAM-34 has been shown to greatly reduce ventricular arrhythmias in human-induced pluripotent stem cell-derived cardiomyocytes by reducing delayed afterdepolarizations and arrhythmic Ca^2+^ transients (8). Previously we found that the expression of KCa3.1 in the LA and RA increased significantly following atrial rapid pacing, while intravenous administration of TRAM-34 7 h later completely inhibited the induction of AF (9). However, acute models are not sufficiently convincing, and the mechanism of KCa3.1 requires additional investigation. In the present study, a prolonged rapid atrial pacing model was established in dogs to elucidate the mechanism of KCa3.1 in cardiac electrophysiology. The results demonstrate that intravenous administration of TRAM-34 three times a day visibly prevented increased vulnerability to AF after 7 days of prolonged rapid atrial pacing. Compared with the pacing group, there was no significant decrease in AERP in the pacing + TRAM-34 group compared with the sham group, while the pacing group displayed a marked decline. These results suggest that the inhibition of KCa3.1 suppressed atrial electrical remodeling.

Previous studies have confirmed that KCa3.1 functions in macrophages, and it has been hypothesized that KCa3.1 channels regulate membrane potential and intracellular calcium hemostasis, in addition to modulation of macrophage polarity (16, 17). This supposition is consistent with the observation that treatment with TRAM-34 reduces the number of activated macrophages in an infarcted hemisphere in a rat model of ischemia/reperfusion injury (stroke) (18). Furthermore, emerging evidence indicates that blocking KCa3.1 significantly reduces the expression of pro-inflammatory genes during macrophage polarization (19). Macrophages can be categorized as either pro-inflammatory (M1, iNOS^+^, Arg1^−^) or anti-inflammatory (M2, iNOS^−^, Arg1^+^), the two extremes of a continuum of phenotypes that macrophages present when exposed to different stimuli (20). M1 macrophages promote inflammation by secreting various inflammatory factors, while M2 macrophages inhibit inflammation by releasing anti-inflammatory mediators (21). The numbers of pro-in?ammatory macrophages, in addition to the levels of pro-in?ammatory cytokines they secrete, including IL-1β and TNF-α, have been shown to be elevated in patients with AF (22–25). In contrast, reduced numbers of anti-in?ammatory macrophages have been observed in AF patients, supporting the premise that AF promotes pro-inflammatory macrophage polarization, cells that additionally cause atrial electrical remodeling (4). In a preceding study, we also found that the sub-type distribution of macrophages in the atria was closely related to the occurrence of atrial fibrillation after acute stroke, the infiltration of M1 macrophages in the atrium increasing significantly 3 days after acute stroke in dogs (26). In the present study, macrophage polarization and up-regulating of KCa3.1 in macrophage was clearly observed in the atrium after 7 days of continuous pacing. To identify pro-inflammatory M1 macrophage and KCa3.1, samples of atrial tissue were incubated with the primary KCa3.1 antibody and CD68 antibody, with cell nuclei stained with DAPI. The results indicate that the number of CD68-positive cells, representing M1 macrophage, had increased markedly in the pacing group compared with the sham or pacing + TRAM-34 groups. The expression of KCa3.1 in macrophage was quantified as the mean optical density. The mean optical density of KCa3.1 in macrophage = Sum of KCa3.1 optical density values in CD68 marked area/CD68 marked area. The results demonstrated the expression of KCa3.1 in macrophage was elevated in pacing group and down-regulated by TRAM-34. The results indicate that TRAM-34 suppresses the pro-inflammatory macrophage polarization promoted by prolonged rapid atrial pacing through down-regulating the expression of KCa3.1 in macrophage. Additional evidence from Western blotting also confirmed that CD68 up-regulation was reduced by TRAM-34. Identical conclusions were obtained from the results of real-time PCR of the expression of iNOS, a biomarker of M1 macrophages, which increased significantly compared with the sham and pacing+TRAM-34 groups, while the expression of Arg-1, a biomarker of M2 macrophages, decreased significantly in comparison with the sham and pacing + TRAM-34 groups. However, we also found that the expression of KCa3.1 in cardiomyocytes increased after atrial pacing. The increased expression of KCa3.1 in cardiomyocytes may have relationship with the atrial electrical remodeling.

In addition to macrophage polarization, the levels of IL-1β, MCP-1, and TNF-α in atrial tissue increased in the AF canine model following prolonged rapid atrial pacing, while reduced macrophage polarization due to the administration of TRAM-34 significantly inhibited the induction of AF and reduced the levels of IL-1β, MCP-1, and TNF-α in the atrium. The results suggest that the effect of TRAM-34 on inflammatory cytokines was related to macrophage polarization. Additional investigation of the underlying molecular mechanisms demonstrated that the p38 MAPK/AP-1/NF-κB signaling pathway was a potential candidate pathway. It has been shown previously that blockade of the p38 pathway increases the expression of M2 macrophage polarization biomarkers and decreases the expression of a number of M1 macrophage polarization biomarkers in RAW264.7 cells compared with control cells, with NF-κB also implicated in proinflammatory macrophage polarization (27, 28). The p38 MAPK signaling pathway has been reported to upregulate KCa3.1 channels via activation of the AP-1 complex (29), the major members of which include the transcription factors c-Fos and c-Jun. In the present study, we found that the expression of the p65 subunit of NF-κB, p38, and c-Fos in atrial myocytes was elevated after prolonged rapid atrial pacing and reduced by TRAM-34. The results indicate that TRAM-34 reduces macrophage pro-inflammatory polarization in the atrium through the p38MAPK/AP-1/NF-κB signaling pathway after prolonged rapid atrial pacing.

### Limitations of the Study

There were several limitations to the present study. Firstly, the study did not confirm the inhibitory effect of TRAM-34 on macrophage polarization in cellular experiments. Previous studies suggest that treatment with TRAM-34 reduces the numnber of activated macrophages in an infarcted hemisphere in a rat model of ischemia/reperfusion injury; however, the effect of TRAM-34 on isolated macrophages requires additional study. Secondly, we found increased pro-inflammatory macrophage polarization in the atrium following continuous pacing, but failed to distinguish between circulating monocytes and cardiac-resident macrophages. Finally, the optimal stimulation parameters for TRAM-34 on macrophages have not been fully determined. In the present study, methods based on previous animal experience were used. Additional study of the effect of TRAM-34 at different concentrations on macrophage polarization is therefore required.

## Conclusions

In the present study, we demonstrated for the first time that the inhibition of KCa3.1 channels reduces the induction of AF after prolonged rapid atrial pacing by reducing the polarization of pro-inflammatory macrophages and the secretion of associated inflammatory factors, probably through the p38 MAPK/AP-1/NF-κB signaling pathway. The results support the hypothesis that inhibition of KCa3.1 suppresses vulnerability to AF through macrophage polarization.

## Data Availability Statement

The raw data supporting the conclusions of this article will be made available by the authors, without undue reservation.

## Ethics Statement

The animal study was reviewed and approved by Institutional Animal Care and Use Committee.

## Author Contributions

All authors listed have made a substantial, direct and intellectual contribution to the work, and approved it for publication.

## Conflict of Interest

The authors declare that the research was conducted in the absence of any commercial or financial relationships that could be construed as a potential conflict of interest.
